# Descriptive Analysis of Emergency Department Patients With Substance Use Disorders As Seen by Peer Recovery Specialists in Philadelphia

**DOI:** 10.7759/cureus.69274

**Published:** 2024-09-12

**Authors:** Kory London, Jokichi Matsubara, Davis Christianson, Jeffrey Gillingham, Megan K Reed

**Affiliations:** 1 Emergency Medicine, Thomas Jefferson University, Philadelphia, USA; 2 Internal Medicine, Temple University Hospital, Philadelphia, USA; 3 Family Medicine, Thomas Jefferson University, Philadelphia, USA

**Keywords:** alcohol use disorder, emergency department, opioid use disorder, peer recovery specialist, polysubstance use, substance use disorder

## Abstract

Background: People with substance use disorders have regular contact with the emergency department (ED). Peer recovery specialists with lived experiences of substance use can provide critical linkages to substance use treatment and other services for patients who use drugs. Patient populations seen by peer recovery specialists remain largely undescribed.

Objective: This paper describes the demographics, substance use patterns, and barriers to treatment of patients seen by peer recovery specialists in an ED in Philadelphia.

Method: A review of patient records about demographics, the reason for ED visits, housing, primary drug of choice, previous treatment, treatment barriers, and urine toxicology screening results was conducted.

Results: Of 228 patients seen between December 1, 2020, and April 8, 2021, those who listed alcohol as their drug of choice (n=56, 24.6%) came to the ED primarily for withdrawal symptoms (n=19, 33.9%). Patients who listed other drug use (n=172, 75.4%) were largely seen for infection (n=57, 33.1%). Polysubstance use was prevalent in patients whose primary drugs of choice were not alcohol. Inpatient treatment was the most common previous treatment previously (n=106, 46.5%) and the most desired treatment preferred for future treatment (n=97, 42.5%). The most common barriers to treatment for patients were medical comorbidities (n=70, 30.7%) and difficulty navigating the healthcare system (n=43, 18.9%).

Conclusions: Patients consulted by peer recovery specialists had distinct demographics, substance use patterns, and perceived goals and barriers to care. These findings highlight the importance of recognizing and treating polysubstance use for people who use drugs and the critical role of peer recovery programs in navigating subsequent care.

## Introduction

The current overdose crisis in the United States is a profound public health issue. Synthetic opioids, mostly analogs of fentanyl, have contributed to increasing mortality rates [1]. Relatedly, emergency department (ED) visits for patients with substance use disorders (SUD) are common [2]. Tools for ED-based SUD intervention are limited; brief intervention alone is not effective [3]. Initiation of buprenorphine, a medication for opioid use disorder (MOUD), is effective at improving rates of subsequent engagement but is underutilized and requires systems to bridge that care [4,5]. Naloxone “to go” is an essential tool to prevent overdose but does not help patients requesting recovery services [6,7].

Health systems have begun to adopt programs of peer recovery specialists to help mitigate barriers to long-term recovery [8]. Peer support increases patient engagement in long-term treatment [9]. Peer support may increase an ED patient’s likelihood of accepting a long-term MOUD referral [10]. In qualitative focus groups, hospital staff found peer specialists to be valuable resources for patients, and a retrospective analysis determined that peer specialists may reduce the time to initiate MOUD [11,12]. The shared lived experience between peers and patients is a resource for developing relationships with patients, particularly in the challenging acute setting of the ED. However, the characteristics and needs of patients who receive peer services in the ED remain largely unknown.

Our study aimed to integrate clinical data with survey data from patient perspectives on drug use, goals, and barriers to treatment to better understand gaps in treatment and areas of focus in the Philadelphia region. Importantly, while peers were funded explicitly to assist patients with OUD, they were utilized to assist patients with all types of SUD.

## Materials and methods

Study population

Thomas Jefferson University Hospital is in Philadelphia, Pennsylvania. The Jefferson Certified Recovery Specialist (CRS) program was developed in partnership with the City of Philadelphia’s Department of Public Health and began service in December 2020 at Thomas Jefferson University Hospital and four other Jefferson hospitals. CRS is the term used to describe a peer recovery specialist/mentor in the Commonwealth of Pennsylvania. CRS consults are recommended for patients with SUD who are open to recovery due to the need for verbal consent by patients for the CRS to engage. ED providers use their discretion in utilizing this service for patients with various clinical presentations to advocate for recovery services and to refer patients to treatment and continued engagement post-discharge. The CRS works with social work colleagues and addiction experts throughout the hospitals, supports patients throughout hospitalizations, and assists with post-acute housing and recovery services. Many of these patients are seen solely in the ED and represent a group with diverse needs and experiences.

Study design

This study is a retrospective descriptive cohort analysis utilizing data from a CRS-delivered questionnaire and medical data taken from the electronic health record, EPIC (EPIC Systems, Madison, WI). Patients engaged by the CRS were given the survey as part of their workflow, but patients completed it at their own discretion. The questionnaire given by the CRS included current housing status, drug of choice, previous treatment modalities tried, and perceived barriers to treatment. Charts of those patients who were surveyed over a four-month interval (December 1, 2020-April 8, 2021) were reviewed for demographic data, reason for ED visit, and results of toxicologic and fentanyl screens. The study was reviewed by the Thomas Jefferson University Institutional Review Board (approval number: 21E.560) and deemed exempt.

The primary reason for the visit for each patient was extracted in the electronic chart review by noting the encounter's chief complaint. These reasons were subsequently grouped into one of these categories: infection, suicidal ideation, intoxication, withdrawal, overdose, request for substance use treatment, and other complaints. These groupings were made after data were collected to better visualize the trends in reasons for ED visits. Two independent reviewers coded the data with discrepancies adjudicated by the senior author. Urine toxicology screens were obtained in the medical chart for the date of service and included screens for benzodiazepines, amphetamines, cocaine, cannabis, barbiturates, opiates (non-fentanyl/methadone), methadone, and fentanyl. Patients with primary alcohol use disorder were distinguished from other drugs of choice for epidemiologic purposes and to highlight the differences in circumstances surrounding ED visits. Patients were determined to have polysubstance use if their drug screens were positive for multiple substances. The authors used the STROBE statement to structure this manuscript [13].

## Results

Between December 1, 2020, and April 8, 2021, CRS were consulted on 228 patients. Demographic characteristics and reasons for visits for patients surveyed by the CRS are listed in Table 1. Cohen’s Kappa test for inter-rater reliability was 0.99. Most patients (n=165, 72.4%) identified as male with a median age of 39 years and were White, non-Latino, or Hispanic (n=151, 66.2%). Reasons for visiting varied depending on primary drug use or alcohol use. Patients with primary drug use (n=172, 75,4%) most often presented with concerns regarding infections (n=57, 33.1%) and requests for substance use treatment (n=26, 15.1%). Conversely, patients with primary alcohol use (n=56, 24.6%) presented with withdrawal (n=19, 33.9%) and intoxication (n=9, 16.1%) as the most common reasons for visit.

**Table 1:** Sociodemographic characteristics of patients consulted by the CRS service for alcohol use and drug use in the TJUH ED in Philadelphia n = number of individuals, % = percentage of individuals in cohort, a = Asian/Native Hawaiian/Pacific Islander, Native American/Alaska Native, and Indian American, b = primary reason for visit that did not fit within above specified categories CRS: Certified Recovery Specialist, TJUH: Thomas Jefferson University Hospital, ED: emergency department

	Drug use, n=172 n (%)	Alcohol use, n=56 n (%)
Age - median	38	43
Gender identity		
Male	124 (72.1)	41 (73.2)
Female	48 (27.9)	15 (26.8)
Race/ethnicity		
White, non-Latino, or Hispanic	127 (73.8)	24 (42.9)
Black, non-Latino, or Hispanic	37 (21.5)	24 (42.9)
Latino or Hispanic	5 (2.9)	7 (12.5)
Other^a^	3 (1.7)	1 (1.8)
Primary reason for visit		
Infection	57 (33.1)	0 (0)
Intoxication	15 (8.7)	9 (16.1)
Overdose	9 (5.2)	0 (0)
Substance use treatment request	26 (15.1)	3 (5.4)
Suicidal ideation	4 (2.3)	2 (3.6)
Trauma	8 (4.7)	3 (5.4)
Withdrawal	12 (7.6)	19 (33.9)
Other^b^	40 (23.3)	20 (35.7)

**Figure 1:**
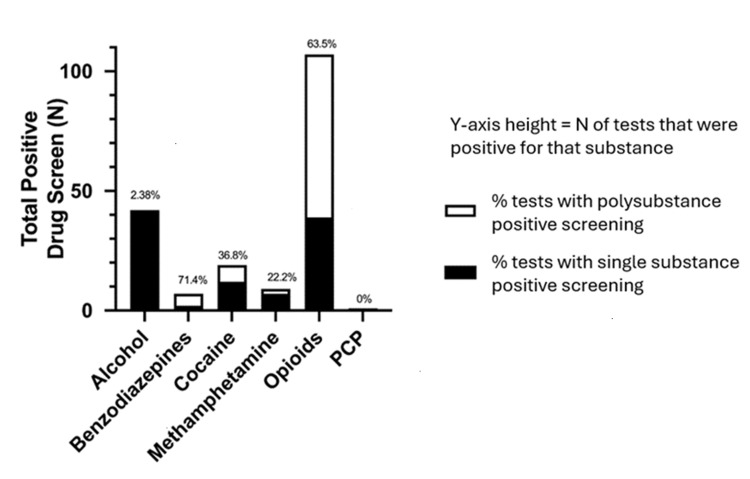
Toxicology screening among patients consulted by CRS service at TJUH stratified by number of positive toxicology screening (and % of those screens that were positive for other substances) Y-axis height = number (n) of tests that were positive for that substance, white segment = % of tests with polysubstance positive screening, black segment = % of tests with single substance positive screening CRS: Certified Recovery Specialist, TJUH: Thomas Jefferson University Hospital, PCP: phencyclidine

Figure 1 describes the associations between polysubstance use and drug of choice. Among those individuals who use opioids, 64 patients (63.5%) were found to be polysubstance use positive on drug screening. Notably, those who use benzodiazepines had the highest rate of polysubstance use at 71.4% (seven patients). Those who listed alcohol and phencyclidine as drugs of choice had the lowest rate of polysubstance use at 2.38% (one patient) and 0%, respectively.

A majority of drug use and alcohol use populations had tried inpatient treatment (59.0% and 60.9%, respectively), and most had goals for additional inpatient treatment (54.9%, 52.2%). The living situation for patients with drug use was only collected for 74% of patients. Among those for whom this information was collected, 25% were undomiciled (Table 2).

**Table 2:** Recovery goals and barriers for patients consulted by the CRS service in the TJUH emergency department in Philadelphia, stratified by drug of choice n = number of individuals, % = percentage of individuals in cohort, a = not collected for drug use and alcohol use (n=40 and n=10, respectively), b = not collected for drug use and alcohol use (n=39 and n=10, respectively), c = not collected for drug use and alcohol use (n=44 and n=10, respectively), d = not collected for drug use and alcohol use (n=39 and n=10, respectively) CRS: Certified Recovery Specialist, TJUH: Thomas Jefferson University Hospital

	Drug use (n, %)	Alcohol use (n, %)
Prior recovery modalities^a^		
Inpatient	78 (59.1)	28 (60.9)
Mutual support meetings	7 (5.3)	1 (2.2)
Outpatient buprenorphine	7 (5.3)	n/a
Outpatient methadone	15 (11.4)	n/a
Outpatient naltrexone	1 (0.8)	0 (0)
Recovery housing	1 (0.8)	1 (2.2)
None	14 (10.6)	12 (26.1)
Other	9 (6.8)	4 (8.7)
Goals for after this visit^b^		
Inpatient treatment	73 (54.9)	24 (52.2)
Mutual support meetings	0 (0)	4 (8.7)
Outpatient buprenorphine	5 (3.8)	0 (0)
Outpatient methadone	8 (6)	n/a
Outpatient naltrexone	1 (0.8)	0 (0)
Recovery housing	2 (1.5)	0 (0)
None	16 (12)	8 (17.4)
Other	28 (21.1)	10 (21.7)
Living situation following discharge^c^		
Family residence	28 (16.3)	11 (19.6)
Friend residence	4 (2.3)	0 (0)
Personal residence	41 (23.8)	23 (41)
Shelter	1 (0.6)	2 (3.6)
Undomiciled	43 (25)	9 (16.1)
Other	11 (6.4)	1 (1.8)
Barriers to treatment^d^		
Difficulty navigating system	32 (24.1)	11 (23.9)
Employment obligations	0 (0)	1 (2.2)
Family obligations	3 (2.3)	1 (2.2)
Medical comorbidities	52 (39.1)	18 (39.1)
Prior poor experiences	6 (4.5)	6 (13)
Stigma from medical system	7 (5.3)	0 (0)
Transportation needs	4 (3)	0 (0)
Other	29 (21.8)	9 (19.6)

Medical comorbidities were the most common barriers to treatment, with 39.1% identifying them in both drug use and alcohol use populations. Additionally, “difficulty navigating system” was the second most common barrier, with 24.1% and 23.9% identifying it in drug use and alcohol use populations, respectively.

## Discussion

In this study, we provide characteristics of patients counseled on substance use by a hospital-based peer-recovery program. Considering the high prevalence of SUDs nationally [14], characterizing the demographics, substance use, and treatment goals and barriers of patients is important to facilitate linkages between patients and support services in the future.

Patients seen were predominantly male, in their 30s (median age 38), as well as non-Hispanic White, relative to other ethnicities. These demographic characteristics are consistent with studies of national data on patients with SUDs presenting in EDs [2].

Previous studies have shown that persons with SUDs are more likely to experience homelessness [15], and in our study, 34% of patients responded to the housing status survey question with either “undomiciled” or “shelter.” Future interventions with patients seen in the ED should consider the high rate of housing insecurity in this population. ED-based peers must be familiar not just with SUD treatment resources but can link patients to resources that address social determinants of health, such as housing.

For patients with primary drug use, infection was the most common reason for visiting the ED. Additionally, “medical comorbidities” were the most common listed reason for barriers to treatment. This is consistent with recent data in the Philadelphia area showing increasing infectious complications of injection drug use [16], and evidence-based methods to reduce infection such as harm reduction counseling and the distribution of syringes, naloxone, and fentanyl test strips are critical areas for future study.

Notably, the number of patients who listed alcohol as their drug of choice (n=56) relative to patients who listed other substances was much lower than typically seen in the ED [17]. There are many possible reasons for this, including providers may be less likely to refer patients with intoxication or alcohol use disorder, or patients with alcohol use disorder may be more likely to decline CRS services. These findings suggest that CRS services may be underutilized for patients with alcohol use disorder, and efforts to increase awareness among providers for this population may be beneficial.

With regards to polysubstance use, most patients with non-alcohol preferences were found to be positive for polysubstance use. Current treatment centers are often geared toward specific substances, but our results show that the high prevalence of polysubstance use should be considered. Additionally, these findings support increased urine screening to better tailor the management of withdrawal and supportive treatments for varied substance use among patients in the hospital.

Lastly, in our study, most patients had tried inpatient treatment for substance use and additionally had goals for inpatient care. This is important in the context of perceived barriers to care, in which “difficulty navigating the system” was the second most common reason for barriers to treatment after medical comorbidities. These findings highlight the role of peer specialists as agents of both engagement and assistance referring through the complex behavioral health system.

Other barriers exist for patients with SUD to access and stay in treatment. These include lack of identification documents and cellphone access, negative stigma associated with MOUD, availability of long-term care and inpatient treatment, insurance-related barriers, and stable recovery housing [11,18,19]. These barriers, which require time-consuming solutions, may contribute to the underutilization of treatment resources for patients. In our study, we found these barriers are common. However, given the limitations of this survey, further qualitative examination of CRS and patients who interacted with the CRS would be beneficial. Given these challenges, holistic systems must be developed to engage and reinforce positive steps towards recovery and safer use.

Limitations

This study faced several limitations. The sample size was limited to 228 patients for a four-month span in the winter of a single academic hospital. The single ED limits the generalizability to other settings or environments. Patients who visited other hospitals or who were seen subsequently (during a more mature phase of the program) may have separate demographics or experiences. This study was limited in its patient population due to provider discretion in CRS consultation, particularly in the alcohol use disorder population. Data collection methods did not capture the full picture of the patients’ experience with drug use, as many patients did not complete the entirety of the survey, leaving some questions unanswered. Additionally, there was no option for multiple drugs in the drug of choice category due to city reporting requirements. There is a potential loss of validity due to the discrepancy between the two data collection sources. The chart review aspect of this work limits the ability to understand patient characteristics that were documented in the chart. It may be that other encounters occurred but were not documented. The questionnaire relied on self-reported data, and the chart review relied on data provided by the medical care teams. The former is limited by recall biases on the part of the patient and the latter by limitations in retrospective review.

## Conclusions

This study characterizes the demographics, substance use, and perceived recovery goals and barriers to care for patients consulted by the newly established peer recovery program in the ED. We found that patients were most often middle-aged, with polysubstance use, seeking inpatient care, and facing barriers to navigating the healthcare system. These findings highlight the importance of recognizing polysubstance use, particularly among patients who use opioids or fentanyl, and the role of programs such as the CRS to aid in navigating inpatient care.
